# Popular Dental Literature

**Published:** 1859-04

**Authors:** S. M. Shepherd


### ARTICLE IV.

*Popular Dental Literature.* By S. M. Shepherd, D. D. S.

As a profession, Dental Surgery is, perhaps, more dependent on popular enlightenment for popular favor than any other known. The evils which it proposes to cure are not generally felt until they become incurable. The most valuable as well as the most effectual remedies are those which are applied before the diseases have given any trouble, hence their nature is never understood by the recipient, nor their value appreciated. An operation for the relief of pain, though simple, if successful, makes a lasting impression in favor of the man who performs it. He who succeeds in extracting an aching tooth with only a moderate quantum of pain, is likely to receive more praise for it than if he had preserved a dozen by skillful plugging. The popularity of a dentist more frequently rests upon his skill as a dentist. He is a good fellow, a polite gentleman, a sociable man, a kind and good hearted man ; or he is very intelligent in reference to matters generally, and of course he must be a good dentist. Would that all professional men possessed these good qualities, but it is proper that their professional reputation should rest upon their professional ability. But how shall the public appreciate that about which they know nothing, and how shall they be enlightened? There are professions in which it would be folly to attempt to explain to the public all the principles necessary to qualify an individual for their practice. I suppose it would be exceedingly difficult for the oculist to describe all the intricacies of his specialty in a manner to interest and profit the general reader; so with the aurist, and so with the surgeon and so with the physician. There is much in the practice of medicine however which may be and has been so described and explained as to enlighten and edify the intelligent general reader; while the great mass of truth connected with it, and which has been developed by scientific research, to the public, stands written in hieroglyphics. It is true also, that in dental science there is much which it is absolutely necessary for the dentist to know, on which it would be both difficult and profitless to discourse to the public. But there is, likewise an immense deal of truth that may be very profitably told. The books that are written for the profession are therefore not calculated for general reading. While there are many passages to be found in almost any well written work calculated to interest, yet they are so divided by other matter of a different kind that the interest is soon lost and the book laid aside. Now suppose all these passages should be collected together; or suppose every dentist should write out a short lecture, treatise or manual, containing only such matter as he might suppose would be understood and appreciated by the public; have it put up in a neat form for general distribution and hand a copy of it to all his visitors, would that not be a scheme to enlighten and instruct, which might be relied on for a profitable result, both to the profession and to the public. Under almost every head in the catalogue of dental diseases might be found something that could be told in a manner to instruct and profit. For instance; a chapter might be written on first dentition for the information of mothers in which they would be deeply interested; and for that chapter alone would prize the little book as a jewel, and store it away in a safe place where it could be come at when wanted. And then the uses of the first teeth; the diseases to which they are subject; the importance of preserving them from destruction before nature authorises their removal; how to determine the time when they should be removed; the difference between the infant molars and the first adult molars—*a very important thing for every body to know, and yet scarcely one in a hundred does know.* Second dentition—how it should be guided. Irregularity—how prevented; and so on through almost the whole catalogue of diseases; all of which might have a chapter that would meet some public demand and answer some anxious inquiry; and as a whole, if well gotten up would be read with a great deal of interest and profit.

I have for the last fifteen years, used as a professional card, or instead of a card, a treatise of this sort, and I find it beneficial in several ways. As a card, it shows my place of business, and makes a good impression as an improved mode of getting up a card. And then it answers the thousand and one questions which are constantly asked the dentist by his patients. As a card, it is not thrown aside and lost as cards are generally, but placed among the books for safekeeping and may be referred to for any matter of information which it may contain, either in your professional advertisement on the cover or anything that may be contained in its pages. It is difficult I know to write such a work; there is a constant temptation to write too much—more than is necessary for the purpose. To draw the line between what would and what would not be understood and appreciated is what few would do in a first attempt. It is difficult furthermore, to write anything on a subject of that sort without the technicalities of the science; and these constitute a formidable obstacle to the reader, and of course militate against the usefulness of such a work.

The writer must call up the language in which he is accustomed to converse with his patients on the various topics introduced—a language that every body understands, so that his reader may be saved the trouble of consulting medical dictionaries for the meaning of terms. Treatises of this sort should be short. It would be folly to write a work that could not be read in an hour's time. Very few persons will read about teeth longer than an hour, and many will tire in less than half the time. It is useless therefore to write more than the generality of persons will read. The beauty of such a treatise is, to have the facts so plain, and so important and striking, and at the same time so short that he who begins to read cannot cease till he reads through. This may be done; I know it is difficult, but strenuous application will accomplish it. I have long been persuaded that if our profession would make an effort in this direction, that a vast amount of good would be accomplished by it. If people knew as much on this subject as they might and ought to know, we should certainly see a different state of things. Is it important to a man that his teeth should be preserved, and is dentistry capable of doing it? Why then does he lose them? simply because he dont know that he can help it. What proportion of the people do so apply and avail themselves of dentistry as to preserve their teeth? I cannot guess but I know from constant observation that it is very small, even in the cities where dentists of undoubted skill are located and may be daily consulted; and of course it is much smaller in the country. It might be urged that dentistry is an *expensive* thing and few comparatively have the *ability* to avail themselves of its benefits. But this will not explain the fact that thousands of the independent—those who live in affluence, and have the ability to provide every comfort that heart can wish, lose their teeth by decay and other diseases to which the teeth are subject. Furthermore, the poor, who *know the benefits of dentistry* will spend the last dollar they have if necessary, to save their teeth,—a fact known to every dentist. The rich are generally more skeptical in matters requiring the expenditure of money than the poor. They seldom spend money at haphazzard—they seldom "buy the pig in the poke," and as dentistry is a dark subject, and full of uncertainty, the less they have to do wtih it the better. It is not because they love money too well, nor because they think too lightly of their teeth that they show so little favor to dentistry; but simply because they do not know that their teeth can be saved. The whole subject is not appreciated because it is not understood. And how otherwise might we expect it to be? There is not a page of popular dental literature to be found in the whole country except here and there—perhaps a half dozen dentists in the United States have published what I have described for their own patrons. It is simply absurd to say that the people dont care anything about it; the people would care about it if they knew about it; and it strikes me that the best way to diffuse information and wake up an interest on this subject is that which I have advised above, viz. let every dentist publish a treatise—his own or some one's else, and keep on hand a constant supply for gratutituous distribution, and hand one of these to every man, woman and child that comes into his office. They will be well received, and many of them read with deep interest; and they will get into the hands of many besides those to whom they are presented, and thus a literature on the subject will be brought within the reach of every body, a literature suited to the capacity and understanding of the people. Dentistry, as a science, has wonderfully advanced within a few years past, and it may be that such a course as I have recommended would have been premature before about this time. The leading doctrines of the science have been sufficiently settled, I think, however, to have justified such a measure ten years ago. If so the profession has not come up to its duty in this respect.

Thus I have written upon what I deem to be a very important subject—important, both to the dentist and to his patrons. Every dentist will bear me witness that an individual who understands the nature of dental operations and also their necessity, will not only submit to them more readily and patiently, but will pay the fee more cheerfully than those who take it at haphazard. And it is right and reasonable that people should have some knowledge of a matter for which they are required to pay, as to its value. Almost the whole amount of information the people possess on the subject is derived from actual experiment; and their testimony is therefore according to the impression made upon the mind, each one for himself, as to whether dentistry is a good thing, or whether it is not a humbug and the whole fraternity of dentists a pack of swindlers. The practice, on the whole, until comparatively a late date would go far to establish the latter conclusion in the absence of better testimony. I think the time has fully come when that testimony should be given, and that by every dentist who offers his professional services to the public.

